# Interest and Expectations for a Herpes Vaccine Among People Diagnosed with Genital HSV 1-2 Infection: Results from an Italian Survey

**DOI:** 10.3390/v16111789

**Published:** 2024-11-18

**Authors:** Lovel Lisac, Angelo Roberto Raccagni, Riccardo Lolatto, Flavia Passini, Chiara Maci, Elena Bruzzesi, Nicolò Moschetta, Antonella Castagna, Silvia Nozza

**Affiliations:** 1 Infectious Diseases Unit, Vita-Salute San Raffaele University, 20132 Milan, Italy; l.lisac@studenti.unisr.it (L.L.); raccagni.angelo@hsr.it (A.R.R.); passini.flavia@hsr.it (F.P.); maci.chiara@hsr.it (C.M.); bruzzesi.elena@hsr.it (E.B.); moschetta.nicolo@hsr.it (N.M.); castagna.antonella1@hsr.it (A.C.); 2 Infectious Diseases Unit, IRCCS San Raffaele Scientific Institute, 20132 Milan, Italy; lolatto.riccardo@hsr.it

**Keywords:** herpes simplex virus, HSV-1, HSV-2, genital herpes, sexually transmitted infection, HSV vaccine, therapeutic vaccine, survey

## Abstract

Genital herpes simplex virus (HSV) is associated with a reduction in quality of life and adverse outcomes. The aim of this study is to assess the interest and expectations for a therapeutic HSV vaccine among individuals diagnosed with genital herpes in Italy. A retrospective survey was conducted at the Infectious Diseases Unit of the IRCCS San Raffaele Scientific Institute, Milan, Italy. The study collected data on demographics, clinical history and interest in HSV vaccination. The results showed that 87.5% of participants were interested in a therapeutic vaccine, with interest higher among younger people and those with frequent genital herpes recurrences. Participants most expected the vaccine to reduce the pain associated with outbreaks, followed by a reduction in the frequency and duration of recurrences. These findings underscore the strong demand for a therapeutic HSV vaccine, especially among those who experience recurrent outbreaks, and highlight the importance of considering patient expectations when developing preventive and therapeutic strategies for genital herpes.

## 1. Introduction

Herpes simplex virus (HSV) is a widespread and prevalent virus that infects millions of people worldwide. It is divided into two primary types: HSV-1, which typically causes oral herpes, and HSV-2, usually responsible for genital herpes [1]. Both virus strains may cause involvement of the oral and genital anatomical areas. Around 3.8 billion people under 50 years of age (64.2%) globally are estimated to have HSV-1 infection, while an estimated 519.5 million people aged 15–49 (13.3%) worldwide have HSV-2 infection [1,2]. A small number of neonates may contract herpes simplex from their mothers during delivery, and this is estimated to occur in 10 out of 100,000 births globally [3]. Even though the infection in the majority of patients remains unrecognized and asymptomatic, oral herpes often results in cold sores, while genital herpes leads to more severe consequences, including painful and recurrent ulcers [1]. Infected individuals are at a higher risk of transmitting or acquiring other sexually transmitted infections (STIs), including HIV [4]. Some reports estimate that individuals infected with HSV-2 have a three-fold higher chance of contracting HIV from their partners already living with the infection [4]. HSV, once contracted, passes through the mucosal epithelial cells of the oral or genital area, and through terminal neurons establishes a lifelong latency within the host [5,6,7,8]. Periodic reactivations, triggered by factors such as stress or immunosuppression, can result in recurrent outbreaks and viral shedding, making HSV a persistent public health concern [5,6,7,8]. Despite the virus’s significant impact on global health, an effective vaccine for HSV remains elusive [9,10,11,12].

Two distinct types of vaccines are employed in managing viral infections—prophylactic and therapeutic vaccines—each differing in the application stage and the immune responses elicited. Prophylactic vaccines are administered prior to viral exposure to prevent infection by inducing a strong humoral immune response, characterized by neutralizing antibodies [13,14]. These antibodies circulate in the bloodstream and neutralize the virus before it can invade cells [13,14]. For instance, prophylactic HPV vaccines provide protection against high-risk HPV strains linked to cervical and other anogenital cancers, as well as strains causing genital warts [13]. Despite its effectiveness, evidenced by sustained seropositivity up to 10 years, vaccine acceptance remains suboptimal [13]. In 2022, approximately 69% of 13-year-olds and 77% of 17-year-olds had received at least one dose of the HPV vaccine, yet only about 50% of younger adolescents completed the full series, highlighting a gap between initiation and completion [13]. In contrast, therapeutic vaccines are innovative treatments aimed at inducing virus-specific T-cell responses to combat existing infections or lesions [13,14]. Unlike prophylactic vaccines, which rely on humoral immunity, therapeutic vaccines target cell-mediated immunity. For example, a modified vaccinia Ankara (MVA) vaccine targeting HPV oncoproteins demonstrated remarkable efficacy in clinical trials, with complete lesion elimination in 90% of treated women and 100% of men [13]. These findings indicate significant potential in reducing the disease burden for individuals already affected by HPV, particularly those with intraepithelial lesions [13]. However, public acceptance of therapeutic vaccines has also been hindered by hesitancy surrounding novel vaccine technologies, a trend exacerbated by scepticism toward mRNA-based COVID-19 vaccines [15]. Efforts to improve education and transparency are essential to enhance trust and adoption of these emerging options [15].

As of today, both a preventive (i.e., to avoid HSV infection) and a therapeutic vaccine (i.e., to avoid recurrences once infection is established and latent) are lacking for both HSV-1 and HSV-2. The pursuit of an HSV vaccine dates back several decades, and various strategies have been explored in the attempt to develop one [16,17,18,19,20,21,22,23,24,25,26,27]. Traditional vaccines have shown limited success in the context of HSV [16,17,18,19,20,21,22,23,24,25,26,27]. The development of a vaccine for HSV is complicated by the virus’s ability to establish dormancy in neuronal ganglia, allowing them to evade the immune system’s natural response to a viral infection [16]. During this latent period, HSV effectively avoids being detected through mechanisms like inhibition of the host’s immune system and down-regulation of major histocompatibility complex (MHC) molecules, important immune components utilized in detecting a foreign microorganism [16]. HSV becomes subjected to immune attack only during its reactivation (the so-called “active phase”), which is the time most patients request help and medication from healthcare professionals [16]. This latent nature of the virus impedes vaccine development, as it requires a vaccine aimed at preventing initial infection as well as controlling or eliminating the virus during reactivation [16,17,18,19,20,21,22,23,24,25,26,27]. This dual challenge of preventing primary infection and recurrence makes HSV a particularly difficult target for vaccine developers.

An important therapeutic vaccine has recently been developed by Glaxo Smith Kline (GSK), named GSK 3943104. This HSV vaccine candidate contained HSV antigens complemented with an adjuvant, designed to stimulate both humoral and cellular immune responses in people already infected with HSV [28,29]. The aim was preventing viral recurrence and reducing the frequency and severity of reactivations [28,29]. The vaccine has shown encouraging results in early-phase clinical trials, particularly in its ability to reduce viral shedding and prevent the reactivation of latent HSV in previously infected individuals [28,29]. Unfortunately, despite very high expectations, during Phase I II clinical trials, GSK 3943104 did not meet its primary efficacy endpoints, meaning it was not successful in significantly reducing lesion outbreaks [28,29]. Subsequently, Phase III was cancelled [28,29]. Despite this, no major safety concerns were observed, and there is strong commitment to continuing the generation of follow-up data, as well as routine safety monitoring [28,29]. This may prove beneficial in the future with the development of new vaccine candidates.

Despite these challenges, the development of an HSV vaccine remains a high priority due to the virus’s widespread prevalence and significant impact on public health [30]. HSV aids in spreading other infections, including HIV, poses immense risks of neonatal infection during delivery, and is a permanent source of physical discomfort and emotional distress in infected individuals, especially young adults [31]. The continued pursuit of an HSV vaccine is vital for addressing these public health concerns, and ongoing research is exploring new frontiers in immunology and virology to overcome the obstacles that have hindered progress thus far. Overall, the development of an effective HSV vaccine has proven to be a complex challenge. The virus’s ability to avoid recognition by the immune system and establishing latency are only some of the obstacles vaccine developers face [9,10,11,12]. However, advancements in vaccine technology offer promising avenues for the future.

Moreover, much evidence exists regarding the stigma surrounding STIs, including genital herpes, and its impact on seeking treatment or vaccines [32,33,34]. For instance, genital herpes has been associated with emotional and psychological challenges, often discouraging individuals from seeking proper diagnosis or treatment [32,33,34]. This stigma is perpetuated by misinformation, social attitudes, and a lack of open communication about sexual health, which can increase the psychological burden of living with HSV and reduce healthcare engagement [32,33,34]. Social stigma might also diminish the quality of life and sexual satisfaction, further emphasizing the need for better societal education and support systems to mitigate these effects [32,33,34]. Disparities, especially when focusing on gender and ethnic minorities, might further exacerbate the gap in access to sexual health services as well as the acceptability and delivery of novel vaccine options [32,33,34].

Indeed, existing therapeutic vaccines, such as the herpes zoster one which has been highly successful in preventing both the incidence and severity of shingles, provide remarkable examples of the potential of these strategies [35,36]. Both genital herpes and herpes zoster infections are associated with painful recurrent outbreaks, and both predominantly affect people with weakened immune systems, including people with HIV [31]. Herpes zoster is most common in later life, when the immune system is naturally weakened, either by chronic illness or old age [31]. The herpes zoster vaccine, particularly the newer recombinant zoster vaccine (RZV), has been effective in reducing both the incidence of shingles and long-term pain, also known as post-herpetic neuralgia, and has shown high uptake after being included in national immunization programs [35,36]. The success of the shingles vaccine offers a promising comparison for herpes simplex vaccination efforts. However, data on patients’ needs and expectations of a potential genital herpes vaccine candidate may still be unclear and require further investigation.

Given these premises, the aim of this study is to evaluate the interest for a therapeutic vaccine against HSV among people diagnosed with genital herpes, to assess the possible characteristics associated with the interest for the vaccine and to describe patients’ demands from a possible vaccine candidate.

## 2. Materials and Methods

This is a retrospective study on people diagnosed with genital herpes in care at the Infectious Diseases Unit of IRCCS San Raffaele Scientific Institute, Milan, Italy, and accessing the HIV outpatient clinic, the STIs walk-in consultation service or receiving HIV pre-exposure prophylaxis (PrEP) prescription. For the purpose of the study aims, a survey was conducted on participants during their routine clinical visits to the Infectious Diseases Unit of IRCSS San Raffaele Scientific Institute, Milan, Italy, in June and July 2024. Patients were subjected to the survey based on a previously confirmed genital herpes simplex diagnosis, both with a positive clinical diagnosis and either a positive real-time PCR genital swab for HSV-1 or HSV-2 (by means of FLOW/Ingenius) or a positive serology for HSV-1 or HSV-2 (IgM antibodies, by means of Liaison XL) between January 2015 and September 2024. Date of survey completion by the patient was defined as date of baseline. None had a recurrence of genital herpes at the time of the survey. Individuals’ and demographic characteristics (including age and gender identity) were collected at baseline. Collected individuals’ characteristics included questions about the reported sexual behaviour and risk factors which might prompt a higher recurrence of genital herpes. Clinical history was collected at baseline, including previous STIs (gonorrhea, chlamydia and syphilis), viral hepatitis B or hepatitis C co-infection, HIV status (and related clinical, immunologic and virologic characteristics), and use of HIV PrEP for people living without HIV. This information was collected at each routine clinical visit by the referring physician as part of routine clinical care. Within the implemented survey on HSV, after counselling of genital herpes and prophylactic and therapeutic vaccines, each participant was asked three questions and categories: (1) How many recurrences of genital Herpes did You experience in the last 12 months? (Answer categorized: (a) none, (b) between 1 and 3, (c) more than 3); this was carried out in order to match with existing clinical data on the number of recurrences experienced and to avoid underreporting; (2) Would You be interested in a therapeutic vaccine against genital Herpes? (Answer options (a) Yes, (b) No); (3) What are Your expectations of this therapeutic vaccine? (Answer options: (a) Reducing the overall number of recurrences, (b) Reducing the overall duration of the recurrence and risk of viral transmission, (c) Reducing the pain at the time of recurrence). After all surveys were collected, the data were computerized in electronic health records and converted into a digital dataset in the following month (September 2024), from which it was analyzed.

Individuals provided written informed consent on the use of their data in scientific analyses and to be included in the Centro San Luigi (CSL) Cohort. The CSL Cohort was approved by the Ethics Committee of the IRCCS San Raffaele Scientific Institute, Milan, Italy (date of approval: 4 December 2017, protocol number 34). Recorded data were anonymized and managed according to good clinical practice. Individuals’ characteristics were retrieved from the CSL Cohort database at date of baseline. The median (quartile 1, quartile 3) and the frequency (%) were used, as appropriate, to describe the individuals’ characteristics and survey results. Mann–Whitney test, Chi-square test and Fischer exact test were used to compare the characteristics of individuals interested or not interested in a therapeutic HSV vaccine, for continuous and categorical variables, as appropriate. A two-sided probability value (*p*-value) < 0.05 was considered statistically significant. Analyses were performed using R, version 4.1.2 (R Foundation for Statistical Computing, Vienna, Austria. URL https://www.R-project.org/ (accessed on 1 September 2024).

## 3. Results

### 3.1. Study Population

Overall, 104 people with a previous diagnosis of genital herpes simplex were surveyed regarding their interest in a herpes simplex vaccination, including 19/104 females (18.3%) and 85/104 males (81.7%). The overall median age was 44.3 years (interquartile range, IQR: 36.1–60.1). In the sample, 67/104 participants were men who have sex with men (MSM—64.4%), 62/104 were people living with HIV (59.6%) and 31/104 were receiving oral HIV PrEP at baseline (73.8%). In the group of people living with HIV, 58/62 (95.1%) of participants had HIV-RNA < 50 copies/mL at baseline, with a median number of years from HIV diagnosis being 22.7 years (IQR: 12.7–31.9). Some participants reported prior episodes of bacterial STIs, including gonorrhea (33/104, 31.7%), chlamydia (28/104, 26.9%) and syphilis (28/104, 26.9%). According to hepatitis C and hepatitis B serology at baseline, 10/104 participants tested positive for hepatitis C (9.62%) and 4/104 tested positive for hepatitis B (3.85%). Over half of the study population indicated that they experience at least one episode of genital herpes recurrence in the 12 months before baseline (between 1 and 3 recurrences: 48/104, 46.2%; more than 3 recurrences: 11/104, 10.6%). Overall, 32 people had previously been taking or were currently receiving daily suppressive genital herpes prophylaxis with valacyclovir; in detail, 14 were still receiving the prophylaxis at baseline. Complete characteristics of included individuals are presented in Table 1.

### 3.2. Interest in Genital Herpes Therapeutic Vaccine

From the sample, 91/104 participants (87.5%) indicated that they were interested in a therapeutic genital herpes simplex vaccine, out of which 16/91 were female (17.6%) and 75/91 were male (82.4%). By looking at the risk factors, it is observable that the majority of the participants interested in the therapeutic vaccine were MSM (58/91, 63.7%), followed by patients exhibiting other risk factors (16/91, 17.6%).

There was a significant statistical difference in the herpes recurrence rates between the two groups, with people interested in the vaccine showing a higher number of recurrences of genital herpes in the previous 12 months (*p* = 0.008). Among the interested participants, 46/91 (50.5%) reported experiencing between 1 and 3 recurrences in the past year, compared to only 2/13 (15.4%) among people who disclosed not being interested. Conversely, 11/13 (84.6%) of those not interested reported no recurrences in the previous 12 months, compared to 34/91 (37.4%) in the interested group. Further, 11/91 (12.1%) of interested participants experienced more than three recurrences of genital herpes in the previous 12 months, while no participants in the not interested group reported having more than three recurrences. Focusing on people currently receiving genital herpes prophylaxis with valacyclovir, 13/14 (92.9%) were interested in vaccination. Moreover, people interested in the genital herpes vaccination were significantly younger than those not interested (interested: 43.1 years, IQR 35.6–57.9 versus not interested: 60.1 years, IQR 44.3–67,1; *p* = 0.019). No other statistically significant differences were found between the groups, for instance, in terms of HIV status and related characteristics, previous bacterial STIs and gender identity (Table 1). Of note, contrary to people interested in the vaccine, among those not interested in a genital herpes vaccination, none was diagnosed with a viral hepatitis co-infection. Full comparisons between people interested or not in a genital herpes vaccination, with relevant statistical results, are presented in Table 1.

### 3.3. Genital Herpes Vaccine Expectations

Finally, participants who reported being interested in the genital herpes vaccination, reported various and uneven expectations. Overall, 42/91 (46.2%) of interested participants stated that they would expect from the vaccine a reduction in the pain associated with genital recurrences, 30/91 (33.0%) a decrease in the overall number of genital recurrences, and 19/91 (20.9%) a reduction in the duration of the genital recurrences.

## 4. Discussion

The results of this survey provide important insights into the individual characteristics, health profiles and expectations of individuals in regard to a potential therapeutic herpes simplex vaccine. The high level of desire for the vaccine, with the vast majority of participants expressing an interest for it, underscores the noteworthy demand for therapeutic solutions to herpes simplex infections. According to the survey, this interest is mostly driven by the chronic and recurrent nature of the infection, particularly among those who experience a larger number of recurrences in the last 12 months.

The majority of participants were male, with most identifying MSM. The MSM population is known to be at increased risk for STIs, which may explain the higher representation in this survey [31].

The high number of people living with HIV in the sample indicates the overlap between HSV and HIV, but also reflects the study center population. Studies have shown that individuals with HIV are more prone to contract HSV and, once infected, suffer frequent and severe HSV recurrences due to immunosuppression. Interestingly, among those not interested in the vaccine, 84.6% were people living with HIV, which suggests that for some individuals, managing multiple chronic infections may lead to different priorities regarding new vaccinations. It is known that immunosuppressed individuals are contraindicated in receiving certain vaccines, mainly live attenuated ones, but if enough information is not given to potential recipients of this vaccine, it may create false conclusions and patients may opt not to take the vaccine [31]. However, we did not find a statistically significant difference in terms of HIV status between people interested or not in the therapeutic genital vaccine.

One of the most interesting findings from the survey is the significant statistical difference in herpes recurrence rates between those interested and not interested in the vaccine. A large proportion of those interested in the vaccine reported experiencing recurrences of genital herpes in the past year. In contrast, the majority of those not interested in the vaccine reported no recurrences. Further, only the individuals in the interested group reported having more than three recurrences, indicating that individuals who suffer frequent outbreaks are much more interested in seeking therapeutic solutions. This is indeed expected and underlines the disease burden faced by individuals experiencing genital recurrences [1]. Rather than infection acquisition or “serostatus”, people are indeed more motivated to receive a therapeutic vaccine due to their personal disease experience and related burden. However, given the possibility of different recurrence rates over years, possibly linked also to external triggers, if a therapeutic vaccine was to be available, it would be key to effectively deliver this kind of preventive strategy to all at-risk individuals.

Another significant finding was the difference in age of patients who are interested versus not interested in the therapeutic vaccine. According to the results of our survey, the median age of participants not interested in a therapeutic vaccine was significantly higher compared to that of those interested. This aligns with the existing literature, which shows that the disease burden, including physical and psychological components, of frequent herpes recurrences (such as pain, embarrassment, and disruption to personal relationships) often leads to a higher demand for treatment options [32,33,34]. Indeed, younger people, especially when focusing on key populations such as MSM, are at increased risk of STIs, including genital herpes [32,33,34]. This is also corroborated by the high number of surveyed individuals who reported a previous bacterial STI. Considering that young MSM with previous STIs are indeed a sexually active population, the high interest in the vaccine is reassuring and might possibly also limit infectiousness and hence viral spread. However, people of increased age might face more severe herpes outcomes, as a result of immune-senescence and existing co-morbidities [1,2,3]. Hence, broad uptake of a potential vaccine candidate among all age groups is paramount.

The survey revealed that those interested in the vaccine had a clear and diverse set of expectations. The most common expectation was that the vaccine would reduce the pain associated with herpes outbreaks. This was followed by reducing the overall number of recurrences and the time duration of each outbreak. These findings indicate that individuals are not only seeking to avoid the number of recurrences but also to avoid the severe symptoms of pain that accompany them. These results, once again, underline the challenges faced by people suffering from genital herpes and the resulting loss in quality of life [1,2,3]. A reduction in the length of recurrence duration, and hence possibly transmissibility, is not stated as of primary interest, as compared to personal improvement of the natural history of the disease.

These expectations can be compared to the ones of the recombinant herpes zoster vaccine. Just as the shingles vaccine alleviates significant patient suffering, a herpes simplex vaccine has the potential to reduce the painful and recurrent nature of genital herpes as well, fulfilling the expectations expressed by the survey participants. The shingles vaccine has been widely accepted, particularly among older adults, due to its effectiveness in preventing painful shingles outbreaks and also reducing the incidence of post-herpetic neuralgia [34,35,36]. One potential barrier to herpes simplex vaccination, however, is the stigma surrounding sexually transmitted infections. Shingles, which is not sexually transmitted, may be perceived differently by the public, allowing for easier acceptance of the vaccine, especially for non-members of key populations. For a herpes simplex vaccine to achieve similar levels of success, it will be important to address stigma and ensure public health advertisements emphasize the potential health benefits of vaccination beyond sexual health, such as reducing chronic pain and improving quality of life, similarly to the challenges faced in the delivery of the HPV vaccine [37].

While this survey indicates a strong interest in herpes simplex vaccination, there are potential barriers to widespread adoption. The stigma associated with genital herpes may deter some individuals from seeking vaccination, even if it is highly effective [32,33,34]. In addition, individuals who are already managing other chronic infections, such as HIV, may deprioritize vaccination. Thus, public health campaigns will need to focus not only on the efficacy of a vaccine candidate but also on addressing misconceptions about the virus and promoting the vaccine as part of a holistic approach to sexual and overall health, by listening to patients’ needs and expectations [38]. Additionally, a significant number of participants in the survey had a history of other STIs, such as gonorrhea and syphilis. This suggests that the target population for a herpes vaccine may have complex health needs, making it important to integrate the vaccine into broader STI prevention programs.

Some limitations need to be acknowledged. The small sample size and the fact that data were collected from specific members of key populations, including people living with HIV, PrEP users, MSM, and clients of a sexual health service, may limit the generalizability of the study results to the overall population. However, we recognize that these represent the core groups most affected by genital herpes and, therefore, those who may be more likely to benefit from a therapeutic genital herpes vaccine candidate.

## 5. Conclusions

The results of this survey highlight a clear demand for a herpes simplex vaccine, particularly among younger adults and those who experience frequent and painful recurrences of the infection, in a cohort consisting mainly of men who have sex with men and people living with HIV. Identification of patients’ needs appears to be pivotal in case of availability of a vaccine in order to effectively prioritize those with the highest need and also to inform vaccine development. Ultimately, the development of a herpes simplex vaccine holds the promise of reducing both the physical and psychological burden of this chronic infection.

## Figures and Tables

**Table 1:** Characteristics of individuals diagnosed with genital herpes according to their interest in a therapeutic genital herpes vaccination.

	Overall	Not Interested	Interested	*p*-Value
	*N* = 104	*N* = 13	*N* = 91	
**Gender Identity (male):**	85 (81.7%)	10 (76.9%)	75 (82.4%)	0.702
**Age (years):**	44.3 [36.1; 60.1]	60.1 [44.3; 67.1]	43.1 [35.6; 57.9]	0.019
**MSM:**	67 (64.4%)	9 (69.2%)	58 (63.7%)	0.699
**PrEP Use:**	31 (29.8%)	2 (15.3%)	29 (31.9%)	1.000
**Living with HIV:**	62 (59.6%)	11 (84.6%)	51 (56.0%)	0.097
HIV-RNA (<50 copies/mL): *	58 (95.1%)	11 (100%)	47 (94.0%)	1.000
Years from HIV diagnosis: *	22.7 [12.7; 31.9]	29.3 [13.2; 32.5]	21.8 [12.7; 31.7]	0.501
**Years of ART:**	19.1 [9.69; 29.1]	29.2 [12.9; 31.6]	18.3 [9.21; 27.9]	0.107
**HCVAb +:**	10 (9.62%)	0 (0.00%)	10 (11.0%)	0.647
**HbSAg +:**	4 (3.85%)	0 (0.00%)	4 (4.40%)	0.864
**Prior episode of gonorrhea:**	33 (31.7%)	4 (30.8%)	29 (31.9%)	1.000
**Prior episode of chlamydia:**	28 (26.9%)	4 (30.8%)	24 (26.4%)	0.744
**Prior episode of syphilis:**	28 (26.9%)	6 (46.2%)	22 (24.2%)	0.106
**Recurrences of genital herpes:** ^ *None* *Between 1 and 3* *More than 3*	45 (43.3%) 48 (46.2%) 11 (10.6%)	11 (84.6%) 2 (15.4%) 0 (0.00%)	34 (37.4%) 46 (50.5%) 11 (12.1%)	0.008
**Daily herpes prophylaxis:** ^	14 (13.5%)	1 (7.7%)	13 (14.3%)	1.000

* For people living with HIV. ^ In previous 12 months. Abbreviations. MSM—Men having sex with men; PrEP—Pre-exposure prophylaxis, HIV—Human immunodeficiency virus, ART—Anti-retroviral therapy, HCVAb—Hepatitis C antibodies, HbSAg—Hepatitis B Surface Antigen.

## Data Availability

Data available upon reasonable request to the corresponding author. Data are not publicly available due to Ethical reasons.

## References

[1] Herpes Simplex Virus World Health Organization, World Health Organization. 13 September 2024. www.who.int/news-room/fact-sheets/detail/herpes-simplex-virus.

[2] Omarova S., Cannon A., Weiss W., Bruccoleri A., Puccio J. (2022). Genital Herpes Simplex Virus—An Updated Review. Adv. Pediatr..

[3] Samies N.L., James S.H., Kimberlin D.W. (2021). Neonatal Herpes Simplex Virus Disease: Updates and Continued Challenges. Clin. Perinatol..

[4] Myhre J., Sifris D. “The Link Between Herpes Simplex Virus and Increased Risk of HIV.” Verywell Health, Verywell Health. 4 October 2024. www.verywellhealth.com/herpes-simplex-virus-hsv-and-hiv-48959.

[5] Zhu S., Viejo-Borbolla A. (2021). Pathogenesis and virulence of herpes simplex virus. Virulence.

[6] Fatahzadeh M., Schwartz R.A. (2007). Human herpes simplex virus infections: Epidemiology, pathogenesis, symptomatology, diagnosis, and management. J. Am. Acad. Dermatol..

[7] Whitley R.J., Roizman B. (2001). Herpes simplex virus infections. Lancet.

[8] Straus S.E., Rooney J.F., Sever J.L., Seidlin M., Nusinoff-Lehrman S., Cremer K. (1985). NIH Conference. Herpes simplex virus infection: Biology, treatment, and prevention. Ann. Intern. Med..

[9] Us D. (2006). Dünden bugüne herpes simpleks virus aşi çalişmalari [Herpes simplex virus vaccine studies: From past to present]. Mikrobiyol. Bul..

[10] Xu X., Zhang Y., Li Q. (2019). Characteristics of herpes simplex virus infection and pathogenesis suggest a strategy for vaccine development. Rev. Med. Virol..

[11] Wijesinghe V.N., Farouk I.A., Zabidi N.Z., Puniyamurti A., Choo W.S., Lal S.K. (2021). Current vaccine approaches and emerging strategies against herpes simplex virus (HSV). Expert Rev. Vaccines.

[12] Whitley R., Baines J. (2018). Clinical management of herpes simplex virus infections: Past, present, and future. F1000Research.

[13] Garbuglia A.R., Lapa D., Sias C., Capobianchi M.R., Del Porto P. (2020). The Use of Both Therapeutic and Prophylactic Vaccines in the Therapy of Papillomavirus Disease. Front. Immunol..

[14] Rappuoli R., Sutter G. (2017). Editorial overview: Preventive and therapeutic vaccines: Vaccination against viral disease-current advances and challenges. Curr. Opin. Virol..

[15] Iqbal S.M., Rosen A.M., Edwards D., Bolio A., Larson H.J., Servin M., Rudowitz M., Carfi A., Ceddia F. (2024). Opportunities and challenges to implementing mRNA-based vaccines and medicines: Lessons from COVID-19. Front. Public Health.

[16] Koelle D.M., Corey L. (2008). Herpes simplex: Insights on pathogenesis and possible vaccines. Annu. Rev. Med..

[17] Bradfute S., Mertz G. (2022). Immune Responses to Herpes Simplex Virus Infection: Implications for Vaccine Development. J. Infect. Dis..

[18] Koelle D.M., Corey L. (2003). Recent progress in herpes simplex virus immunobiology and vaccine research. Clin. Microbiol. Rev..

[19] Koelle D.M. (2006). Vaccines for herpes simplex virus infections. Curr. Opin. Investig. Drugs.

[20] Gottlieb S.L., Giersing B., Boily M.C., Chesson H., Looker K.J., Schiffer J., Spicknall I., Hutubessy R., Broutet N. (2019). WHO HSV Vaccine Impact Modelling Meeting Working Group. Modelling efforts needed to advance herpes simplex virus (HSV) vaccine development: Key findings from the World Health Organization Consultation on HSV Vaccine Impact Modelling. Vaccine.

[21] McAllister S.C., Schleiss M.R. (2014). Prospects and perspectives for development of a vaccine against herpes simplex virus infections. Expert Rev. Vaccines.

[22] Wise T.G., Pavan P.R., Ennis F.A. (1977). Herpes simplex virus vaccines. J. Infect. Dis..

[23] Mindel A. (1984). Herpes vaccine. Br. J. Vener. Dis..

[24] Hall M.J., Katrak K. (1986). The quest for a herpes simplex virus vaccine: Background and recent developments. Vaccine.

[25] Coleman J.L., Shukla D. (2013). Recent advances in vaccine development for herpes simplex virus types I and II. Hum. Vaccines Immunother..

[26] Gottlieb S.L., Giersing B.K., Hickling J., Jones R., Deal C., Kaslow D.C. (2019). HSV Vaccine Expert Consultation Group. Meeting report: Initial World Health Organization consultation on herpes simplex virus (HSV) vaccine preferred product characteristics, March 2017. Vaccine.

[27] Dix R.D. (1987). Prospects for a vaccine against herpes simplex virus types 1 and 2. Prog. Med. Virol..

[28] Healthcare, GlobalData “As GSK Discontinues HSV Vaccine, Opportunities Remain for Moderna and BioNTech.” Clinical Trials Arena, Clinical Trials Arena. 25 September 2024. www.clinicaltrialsarena.com/analyst-comment/gsk-hsv-vaccine-moderna-biontech/?cf-view.

[29] GSK’s Experimental Herpes Vaccine Fails to Meet Main Goal in Trial|Reuters. Reuters.Com, Reuters. 11 September 2024. www.reuters.com/business/healthcare-pharmaceuticals/gsks-experimental-hsv-vaccine-fails-meet-main-goal-trial-2024-09-11/.

[30] Ferenczy M.W. (2007). Prophylactic vaccine strategies and the potential of therapeutic vaccines against herpes simplex virus. Curr. Pharm. Des..

[31] Rubin L.G., Levin M.J., Ljungman P., Davies E.G., Avery R., Tomblyn M., Bousvaros A., Dhanireddy S., Sung L., Keyserling H. (2014). 2013 IDSA clinical practice guideline for vaccination of the immunocompromised host. Clin. Infect. Dis..

[32] Lee A.S.D., Cody S.L. (2020). The Stigma of Sexually Transmitted Infections. Nurs. Clin. N. Am..

[33] Bobrow M. (2016). Full Disclosure: Herpes Stigma and Communication Practices among HSV+ Individuals. Clark Digital Commons.

[34] Priest N. (2024). HSV+ and Proud: Does Illness Identity Mediate the Relationship Between Genital Herpes Stigma and Well-Being?. Ph.D. Thesis.

[35] Lautenschlager S. (2003). Herpes simplex und Varizella-Zoster-Virus-Infektionen [Herpes simplex and varicella zoster virus infections]. Ther. Umsch..

[36] Zerbo O., Bartlett J., Fireman B., Lewis N., Goddard K., Dooling K., Duffy J., Glanz J., Naleway A., Donahue J.G. (2024). Effectiveness of Recombinant Zoster Vaccine Against Herpes Zoster in a Real-World Setting. Ann. Intern. Med..

[37] Iova C.F., Daina L.G., Daina M.D., Ghitea T.C. (2024). The Effectiveness of Interventions Targeting Adolescents in HPV Vaccination-A Scoping Review. Medicina.

[38] Rupp R., Bernstein D.I. (2008). The potential impact of a prophylactic herpes simplex vaccine. Expert Opin. Emerg. Drugs.

